# Introducing Crack–Termination Sites to Improve the Resistance of Polycarbonate on Environmental Stress Cracking

**DOI:** 10.3390/polym18172042

**Published:** 2026-08-23

**Authors:** Minjian Ma, Qian Huang, Peitao Wang, Junwei Ai, Huiqiang Liang, Liang Yu, Minle Peng, Yin Cen

**Affiliations:** 1 Key Laboratory of Polymer Materials Engineering, Polymer Research Institute, Sichuan University, Chengdu 610065, China; maminjian@kingfa.com.cn (M.M.); qianhuang@scu.edu.cn (Q.H.); 2 Kingfa Science and Technology, Co., Ltd., Guangzhou 510663, China; wangpeitao@kingfa.com.cn (P.W.); aijunwei@kingfa.com.cn (J.A.); lianghuiqiang@kingfa.com.cn (H.L.); yuliang@kingfa.com.cn (L.Y.); pengminle@kingfa.com.cn (M.P.); 3 School of chemistry and chemical engineering, Huazhong University of Science and Technology, Wuhan 430074, China

**Keywords:** polycarbonate, environmental stress cracking, crack termination sites, critical strain rate

## Abstract

Environmental stress cracking (ESC) severely limits the long-term reliability and engineering application of polycarbonate (PC)-based materials. In this work, a universal strategy is proposed to enhance the ESC resistance of PC by introducing crack-termination sites, enabling efficient suppression of crack propagation without compromising the intrinsic mechanical properties of PC. A quantitative evaluation framework based on a constant-strain method is established, and the critical strain rate (η) is defined as a key parameter for assessing ESC behavior under chemo-mechanical coupling. Systematic experiments reveal that chain entanglements, rubbery phases, and crystalline region can effectively increase η value of PC-based materials, demonstrating their superior crack-termination efficiency. Notably, the β-crystalline phase in PBT and microcrystalline domains induced by ethylene–acrylate copolymer tougheners are identified as the most efficient crack-termination structures, providing continuous energy-dissipation pathways and effectively halting crack propagation. This work not only establishes a practical and quantitative approach for evaluating ESC performance but also provides an integrated material-modification strategy. The proposed concept of crack-termination sites offers new insight into the development of high mechanical performance and ESC resistance PC-based polymer systems for advanced industrial applications.

## 1. Introduction

Polycarbonate (PC), a high-performance engineering thermoplastic, has been widely utilized in electronics, automotive components, medical devices, and optical materials due to its exceptional mechanical strength, transparency, thermal resistance, and dimensional stability [1,2,3]. However, PC has a rigid molecular structure, making it highly prone to developing significant orientation and cooling-induced internal stress during injection molding. Therefore, the long-term reliability of PC-based materials in harsh environments are significantly compromised by environmental stress cracking (ESC), a catastrophic failure mechanism triggered by the synergistic effects of chemical media (e.g., organic solvents, greases, or surfactants) and mechanical stress [4,5]. This phenomenon induces localized microcrack initiation and rapid propagation, critically limiting the application of PC-based materials in demanding industrial scenarios [6,7,8].

The intrinsic mechanism of ESC derives from the weakening of intermolecular interactions in PC through chemical media-induced surface adsorption, swelling, or localized plasticization. The weakened intermolecular forces within PC may be insufficient to counteract its high level of internal stress, promoting craze formation and crack growth at stress-concentrated regions [9,10,11]. Conventional strategies to mitigate ESC focus on optimizing molecular architecture of PC, such as introducing branched or cross-linked units or enhancing chemical resistance through alloying with high content of ABS or PBT [12]. Nevertheless, these approaches often sacrifice inherent advantages of PC, such as high-impact resistance, while struggling to balance ESC resistance with processability and mechanical performance [13].

Recent advances in nanocomposite modification offer promising alternatives, such as employing nanoparticles to hinder crack propagation through interfacial reinforcement. However, challenges persist in achieving homogeneous dispersion of nanofillers and robust interfacial compatibility [14,15,16]. Additionally, physical modifications like surface coatings or plasma treatments usually have high cost and provide only transient chemical resistance, failing to meet long-term service requirements [17,18]. Therefore, developing a modification strategy that preserves intrinsic properties of PC and PC-based polymer materials while ensuring durable ESC resistance remains a critical challenge in polymer science.

Extensive efforts have been devoted to improving the ESC resistance of polycarbonate (PC)-based materials. However, intrinsic constraints, such as the inherent high internal stress and reactive ester groups of PC, remain unmodifiable, making it challenging to entirely prevent ESC under combined chemo-mechanical loads [19]. Consequently, suppressing crack propagation within the matrix emerges as a viable pathway to enhance ESC resistance of PC-based materials [20]. Inspired by this idea, this work establishes a systematic quantification framework for the evaluation of ESC performance, enabling hierarchical selection of crack-termination sites [21]. By optimizing the crack-termination sites, a holistic improvement in ESC resistance of PC-based polymer materials is achieved while preserving the intrinsic advantages of the materials.

## 2. Materials and Methods

### 2.1. Materials

PC 2220 (Mn = 22,000, PDI = 1.54) and PC 2100 (Mn = 30,000, PDI = 1.53) was supplied by Wanhua Chemical Co., Ltd. (Yantai, China, https://www.whchem.com), PC 7030PJ (Mn = 48,000, PDI = 1.37) was supplied by MITSUBISHI Chemical Co., Ltd. (Kitakyushu, Japan, https://www.mcgc.com), ABS (ABS 8434) was provided by Gaoqiao petrochemical Co., Ltd. (Shanghai, China, http://sgpc.sinopec.com), and PBT (PBT GX121) was supplied by Yizheng Chemical Fiber Co., Ltd. (Yizheng, China, http://ycfc.sinopec.com); BDP (FP-600) was supplied by Adeka (Shanghai, China, https://adeka.cnreagent.com); MBS (EM500) was provided by LG Chem. (Yeosu, Korea, https://www.lgchem.com); and EMA (Elvaloy AC resin 1125) that contained 32% methyl acrylate and 8% glycidyl methacrylate according to manufacturer specifications was obtained from Arkema Investment Co., Ltd. (Shanghai, China, https://www.arkema.cn). Poly(ethylene-co-butyl acrylate-co-glycidyl methacrylate) (Elvaloy PTW copolymer) was obtained from Dow chemical company (Midland, Michigan, USA, https://cn.dow.com). Antioxidant was purchased from BASF Co., Ltd. (Shanghai, China, https://www.basf.com). The test solvents used to evaluate the ESC resistance of the materials included sunscreen lotion (Banana Boat) and acetic acid (99.9%).

### 2.2. Sample Preparation

In this work, the PC-based alloys were prepared by a twin-screw extruder (Nanjing Only Extrusion Machinery Co., Ltd., Nanjing, China) with a rotating speed of 80 rpm. The extrusion temperatures were set to 220 °C (hopper, region I), 225 °C (region II), 230 °C (region III), 235 °C (region IV–IX), and 225 °C (extrusion head, region X), respectively. Before injection molding, the as-prepared blends were dried in an air-blowing oven for 6 h at 80 °C. Then, the preparation of the standard samples for mechanical performance tests was performed on an injection-molding machine (Ningbo Haitian Plastic Machinery Group Co., Ltd., Ningbo, China), and the melt temperature was 260 °C. The mold temperature was kept at 60 °C.

### 2.3. Characterization

Thermal properties were determined by DSC; ~10 mg of samples was heated and cooled twice in a temperature range of 0–350 °C with a heating/cooling rate of 10 °C/min.

The notched Izod impact test was carried out using a Zwick impact tester (ZwickRoell GmbH & Co. KG, Ulm, Germany) according to ASTM D256, using a 2.75 J hammer. The tensile performance was performed with a crosshead speed of 50 mm/min on a CMT4204 testing system (Shenzhen Suns Technology Co., Ltd., Shenzhen, China) according to ASTM D638. All samples were pretreated for more than 48 h at 25 °C and 50% relative humidity for further testing and characterization, and the average value was calculated using at least five specimens. The vertical burning test was performed on a CZF-5 instrument (Jiangning Analysis Instrument Company, Nanjing, China) according to UL-94. The specimen size was 127.0 × 13.0 × 1.5 mm^3^.

**Figure 1:**
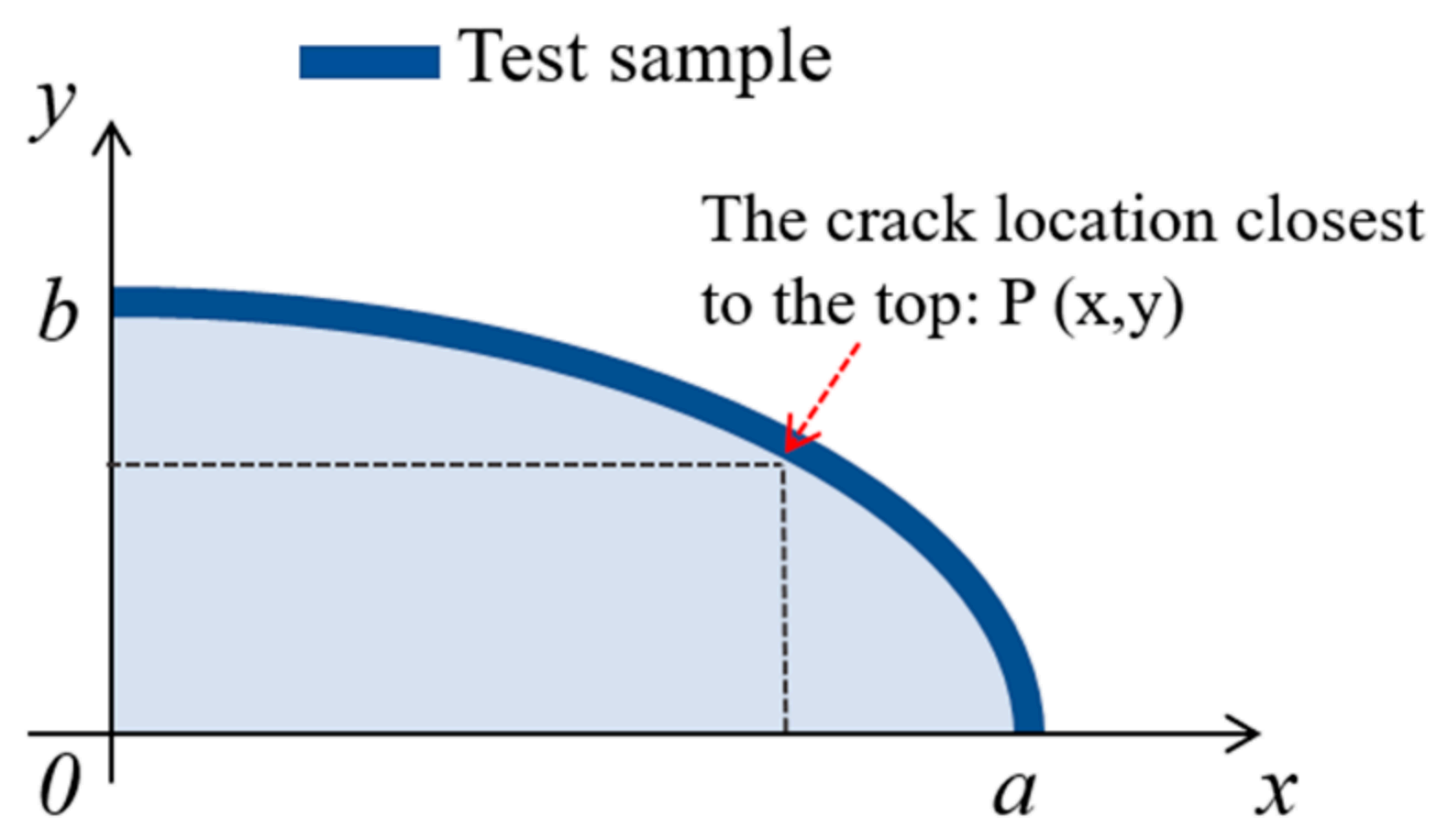
Schematic diagram of the apparatus for testing the ESC resistance of specimens.

The ESC of samples is evaluated with constant strain method according to ASTM D543. Rectangular strip specimens with dimensions of 150 × 15 × 1.5 mm^3^ were fixed onto a quarter-elliptical fixture, as shown in Figure 1. After uniformly applying the test solvent onto the specimen surface, the specimens were wrapped with a sealing film and placed in a constant temperature and humidity environment for 168 h (the solvent was replenished on the sample surface every 24 h). Subsequently, the crack morphology on the specimen surface was observed, and the minimum mold scale corresponding to the crack position was recorded. The critical strain rate of the specimen after 168 h of exposure to the solvent was then calculated according to the following equations:

A Cartesian coordinate system was established with the center of the quarter-elliptical fixture as the origin and its major and minor axes as the x- and y-axes, as shown in Figure 1. The radius of curvature R at a crack location P (x, y) is expressed as follows:

(1) $$R=\frac{{{(a}^{4}y^{2}+b^{4}x^{2})}^{\frac{2}{3}}}{a^{4}b^{4}}$$

where a and b are the lengths of the major and minor axes of the elliptical fixture, respectively. The critical strain η at the crack location is given by the following equation:

(2) $$\eta =\frac{0.5d}{R+0.5d}$$

where d is the thickness of the specimen, which was 1.5 mm in this work. It should be noted that no measurable change in sample thickness was detected (the thickness remained 1.5 ± 0.02 mm) after the ESC test, confirming that solvent-induced swelling had a negligible effect on the thickness parameter used in the strain calculation.

An accelerated stress-releasing test was also designed using injection-molded pillars with embedded copper nuts. The samples prepared with the PC-based materials in this work were totally immersed in acetic acid for 168 h, with the container sealed and maintained at an ambient temperature of 23 °C and 50% relative humidity. The development of surface cracks over time was observed and recorded using a Leica optical microscope (Leica Microsystems, Shanghai, China).

## 3. Results and Discussion

**Figure 2:**
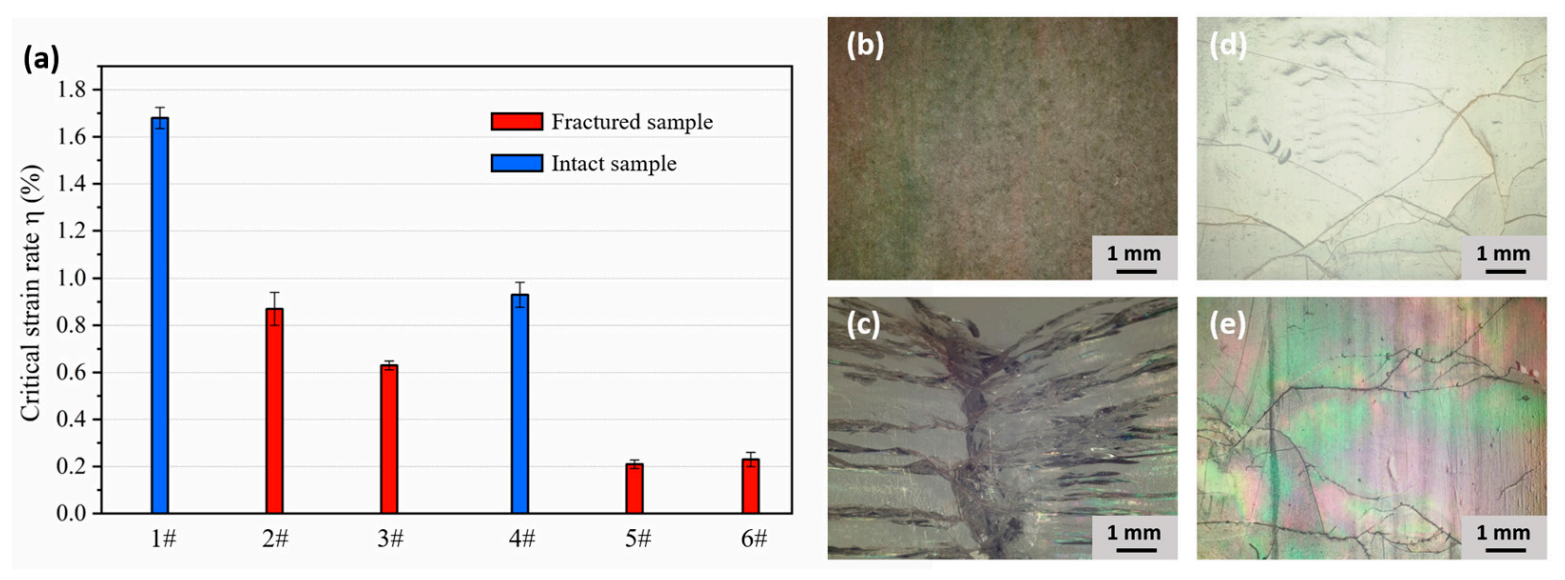
(**a**) The critical strain rate (η) of different samples upon exposure to sunscreen lotion for 168 h; (**b**–**e**) the crack morphology of the Sample 1#, 3#, 4# and5# after the environmental stress crack test (observed at the position corresponding to a strain rate of 1.5%).

PC has relatively rigid molecular chains and a high level of internal stresses (including both flow-induced orientation stress and thermal stress arising from inhomogeneous cooling), inevitably developed during injection molding. Therefore, a proposed mechanism for the ESC of PC is that, upon exposure to chemical agents, solvent-induced swelling reduces the intermolecular interactions within the polymer matrix. When these weakened interactions are no longer sufficient to balance the internal stresses, ESC is prone to occurring. A critical challenge in improving ESC resistance of polycarbonate (PC) lies in establishing an efficient quantitative framework to characterize its ESC behavior. While extensive research has focused on phenomenological observations of ESC, current studies predominantly rely on subjective analysis (visual crack inspection or ranking systems), lacking universally applicable metrics to objectively evaluate material performance under chemo-mechanical coupling conditions [22,23]. To address this gap, we developed a standardized quantitative protocol based on the constant strain method [24]. As illustrated in Figure 1, a quarter-elliptical fixture was designed to impose a controlled strain gradient along the specimen, enabling systematic mapping of strain rate distribution across predefined regions. This configuration allows precise determination of the critical strain rate (η), defined as the threshold strain rate at which solvent-induced crack initiation occurs within a specified exposure duration [25]. The η parameter serves as a quantitative descriptor of ESC resistance, which can distinguish the differences in ESC resistance among different materials (Sample 1#: high-viscosity PC; Sample 2#: medium-viscosity PC; Sample 3#: low-viscosity PC; Sample 4#: medium-viscosity PC with 10 wt.% ABS; Sample 5#: medium-viscosity PC with 10 wt.% BDP; and Sample 6#: medium-viscosity PC with 10 wt.% ABS and 10 wt.% BDP), as demonstrated in Figure 2a (with sunscreen as the test solvent). Complementary analysis of crack morphology under 1.5% strain rate (Figure 2b–e) further reveals distinct ESC response mechanisms influenced by material composition. According to the η results and crack morphology, resin viscosity of PC, ABS alloying, and phosphorus-based flame retardant (bisphenol-A bis(diphenyl phosphate), BDP) will all significantly affect the resistance of PC on ESC.

High-viscosity PC resins (η = 1.68%) exhibited a 1.05% increase in η compared to low-viscosity PC (η = 0.63%). This enhancement stems from the elevated molecular weight and extended polymer chains in high-viscosity PC, which promotes denser physical entanglement networks [26]. The viscosity variations showed negligible impact on solvent swelling kinetics and intrinsic stress levels (confirmed by dynamic mechanical analysis; Figure S1, Supporting Information), the improved ESC resistance directly correlates with entanglement density. These topological constraints act as effective crack-termination sites by dissipating strain energy through chain slippage and restricting crack development, as evidenced by the long and interconnected cracks in low-viscosity PC (Figure 2b,c). However, a high proportion of high-viscosity PC can impair the molding processability of PC-based materials and compromise the surface appearance of the final products. Therefore, improving ESC resistance solely by increasing PC viscosity is of limited practical feasibility.

Incorporating a small amount of ABS (10 wt.%) into PC only slightly increased the η (0.93%) of the material; however, the degree of cracking was significantly mitigated, as the sample changed from a fractured state after testing to exhibiting only non-penetrating surface cracks. The polybutadiene (PB) rubber phase in ABS (~25% content) functions as a nano-scale crack-termination site, which will generate microvoids that blunt crack tips and redistribute stress fields. Moreover, the ABS matrix maintains good compatibility with PC, ensuring homogeneous dispersion and the PC/ABS interfaces deflect crack propagation paths, as evidenced by tortuous fracture surfaces with multiple crack bifurcations (Figure 2d).

Incorporation of 10 wt.% BDP reduced η by 0.66%, severely compromising ESC performance. The phosphoric ester structure of BDP induces the following dual detrimental effects: (i) plasticization—BDP molecules intercalate between PC chains, reducing intermolecular cohesive energy density via dipole–dipole interactions; and (ii) phase incompatibility—low molecular weight of BDP (Mw ≈ 500 g/mol) and aliphatic character promote localized phase separation, creating weak interfaces that accelerate solvent permeation [27]. Figure 2e reveals continuous crack propagation through BDP-contained PC, validating its role as a stress concentrator.

**Figure 3:**
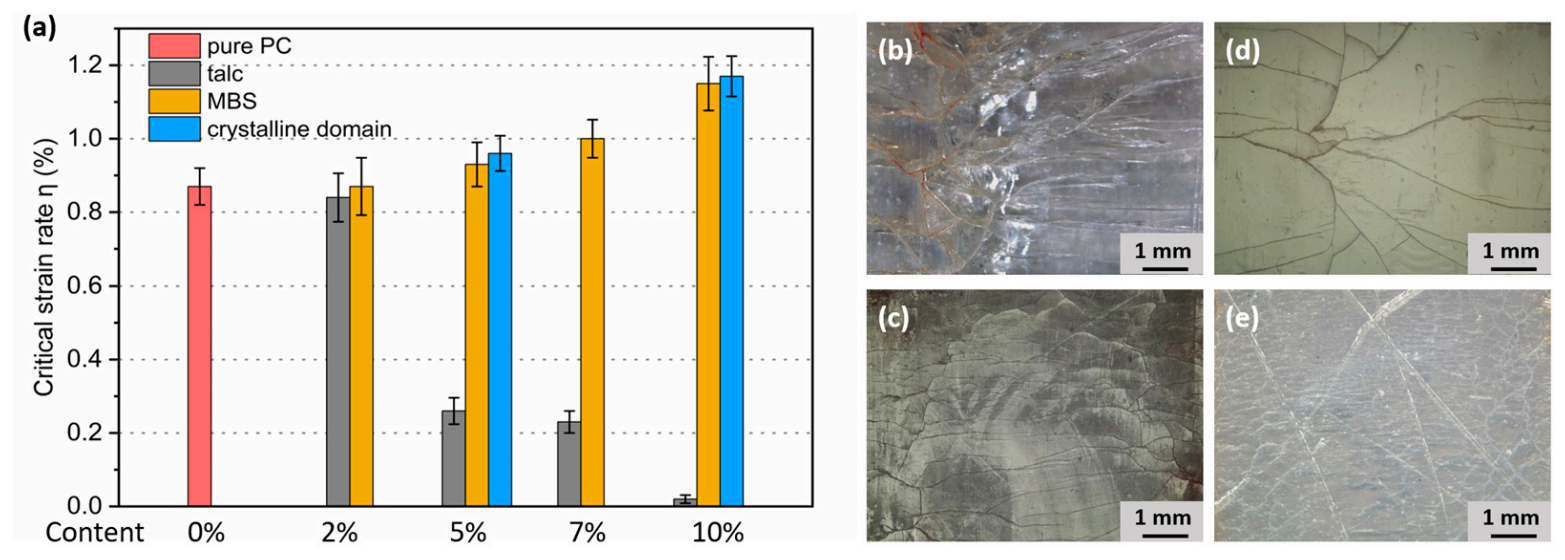
(**a**) The critical strain rate (η) of PC samples with different content of inorganic fillers, toughener and crystalline phase; (**b**–**e**) the surface crack morphology of samples containing different types of crack-termination sites, observed at the position corresponding to a strain rate of 1.5% after the environmental stress crack test: (**b**) medium-viscosity PC; (**c**) medium-viscosity PC with 10 wt.% talc; (**d**) medium-viscosity PC with 10 wt.% MBS; (**e**) medium-viscosity PC with 30 wt.% PBT (with a crystallinity of ~10%).

Given the inherent challenges in mitigating internal stress and modifying the surface chemistry or acid/base sensitivity of PC resin, the strategic introduction of crack-termination sites emerges as a viable pathway to enhance ESC resistance of PC-based materials. While physical entanglements (high-viscosity PC) and rubber phase (ABS alloying) demonstrate effectiveness as the crack-termination sites, alternative crack arrestors, such as inorganic fillers, toughener and crystalline domains, are also studied in this work, as systematically compared in Figure 3a.

Using inorganic particulates (e.g., talc, TiO_2_) as crack-termination sites will also introduce stress concentration sites due to their poor interfacial compatibility with the PC matrix. Despite their high modulus and theoretical potential to deflect cracks, agglomerated fillers act as micron-scale defects that amplify local strain fields. As shown in Figure 3a, PC composites with 10 wt.% talc exhibit a ~100% reduction in η compared to pure PC, accompanied by accelerated crack propagation along filler-matrix boundaries. Regression analysis was also performed on the relationship between talc content and η. The coefficient of determination (R^2^) of the regression model is 0.87 (F = 13.25; *p*-value = 0.035), indicating a strong correlation between the decrease in η and the increase in talc content. This degradation correlates with increased void density and reduced toughness.

In contrast, methyl methacrylate-butadiene-styrene (MBS), which is widely applied in PC-based materials as impact modifier, can obviously enhance ESC resistance, as evidenced by the increased η with the addition of MBS [28]. The rubber phase in MBS can dissipate strain energy through deformation. Moreover, nanoscale dispersion of MBS minimizes stress concentrations. Increasing MBS content from 0% to 10 wt.% elevates η from 0.84% to 1.15%, and the coefficient of determination (R^2^) of the regression model is 0.95 (F = 41.14; *p*-value = 0.002). The superior performance also stems from good interfacial bonding via the grafted PMMA shell of MBS, which maintains matrix compatibility while enabling efficient stress transfer.

Crystalline regions outperform both rubber phases and inorganic fillers in the enhancement of ESC resistance, attributed to the effect of semi-crystalline structure of PBT [29]. The η of PC/10 wt.% PBT alloy (with a crystallinity of ~5%) is 0.96%, and η of PC/30 wt.% PBT alloy (with a crystallinity of ~10%) is 1.17%. The transesterification reaction between the PC and PBT phases ensures excellent interfacial compatibility within the alloy matrix [30]. The crystalline regions in the PBT phase demonstrate superior crack-termination capabilities, effectively arresting crack propagation through their ordered molecular structure. Experimental observation reveals that the PC/PBT sample only exhibited micro-scale, grid-like surface fissures after the ESC test, as shown in Figure 3e.

To elucidate the superior crack-termination capability of crystalline domains, the role of crystal polymorphism in PC/PBT alloys was further investigated. Since both PC and PBT contain ester groups, transesterification reactions occur between them during high-temperature processing [31,32]. This reaction will improve the compatibility of PC and PBT at elevated temperature, but excessive reaction rate will also concurrently disrupt the crystallization behavior of PBT phase [33]. Trace amounts of transesterification inhibitors (less than 0.5 wt.%), such as sodium dihydrogen phosphate, are routinely incorporated in practical PC/PBT alloy formulations. These additives serve to moderate reaction intensity of transesterification, thereby preventing excessive degradation of mechanical properties caused by molecular chain scission. The transesterification reaction between PC and PBT affects the crystalline structure of PBT, which will further influence the ESC resistance of the materials.

**Figure 4:**
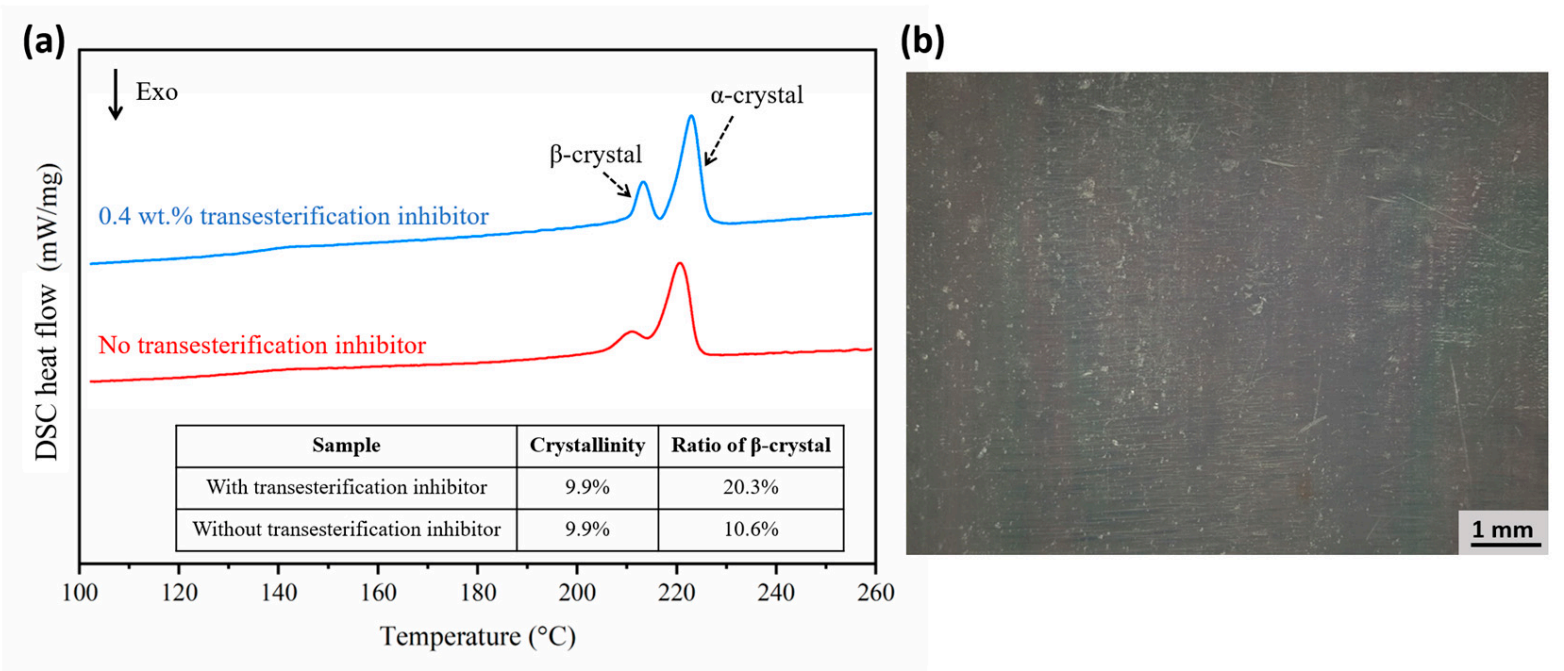
(**a**) DSC curves of the PC/PBT samples with and without transesterification inhibitors, with the inset table listing the crystallinity and the ratio of α and β crystals calculated from the integrated areas of the melting peaks; (**b**) the surface crack morphology of the PC/PBT alloy with transesterification inhibitor, observed at the position corresponding to a strain rate of 1.5% after the ESC test.

As evidenced by DSC curves of PC/PBT alloys (30 wt.% PBT) with and without transesterification inhibitors in Figure 4a, the inclusion of inhibitor shows negligible impact on the overall crystallinity of the PBT phase (~10%). However, it exerts a pronounced regulatory effect on the α-/β-crystal form ratio. Specifically, the inhibitor facilitates preferential retention of β-crystal configurations during high-temperature processing, resulting in alloy with elevated β-phase content. The strain-rate-controlled ESC evaluation reveals obvious morphology differences (Figure 3e and Figure 4b). Inhibitor-free sample exhibits interconnected grid-like crack networks under chemical exposure, whereas inhibitor-modified counterpart displays sparse, non-propagating microcracks. This disparity confirms superior crack-termination efficiency of β-crystals compared to α-crystals at equivalent crystallinity level, which is primarily attributed to the advantageous crystalline structure of the β-phase, thereby endowing the material with superior ESC resistance [34].

**Figure 5:**
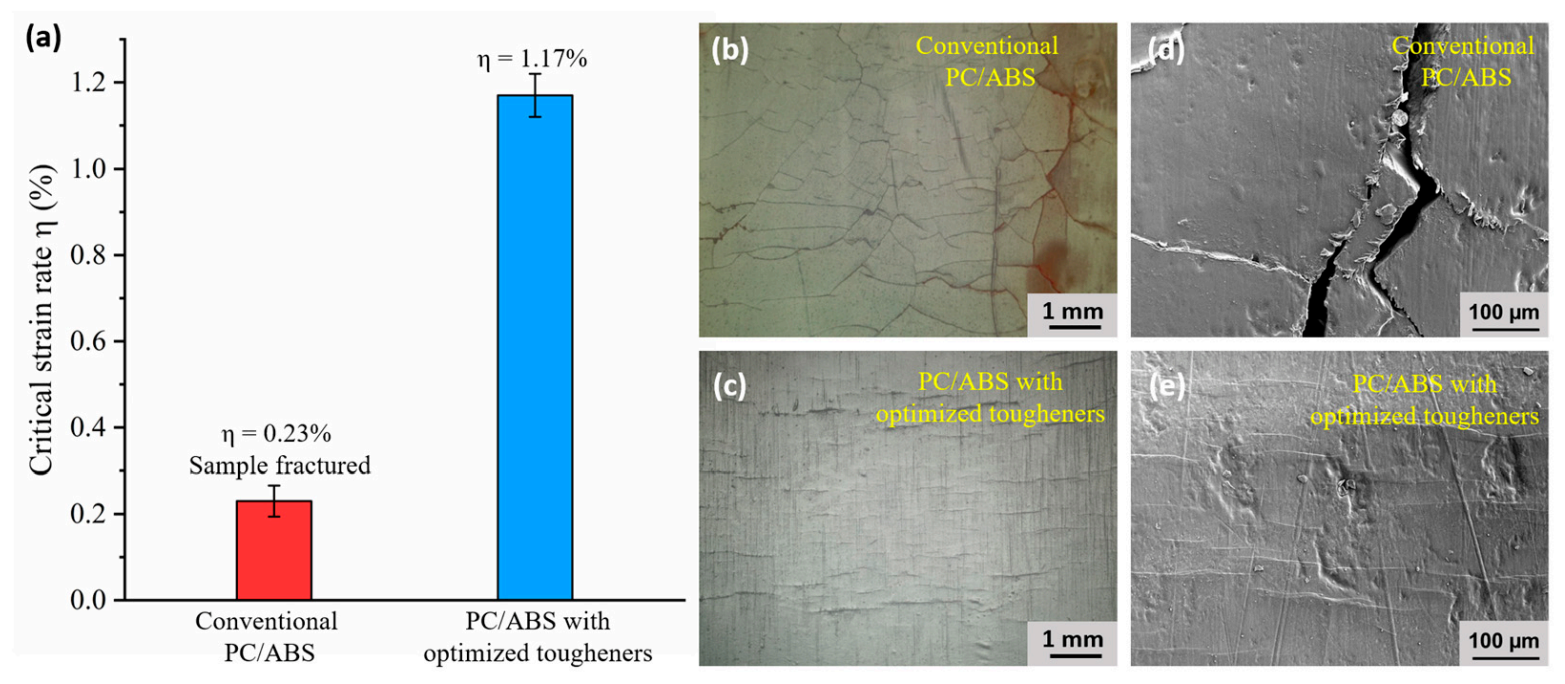
(**a**) Comparison of η value of a conventional flame-retardant PC/ABS alloy with 7 wt.% MBS as toughener and the flame-retardant PC/ABS alloy with an optimized toughener system; (**b**,**c**) the surface crack morphologies of two samples at a strain rate of 1.5%. (**d**,**e**) The SEM images of the surface crack of two samples.

**Figure 6:**
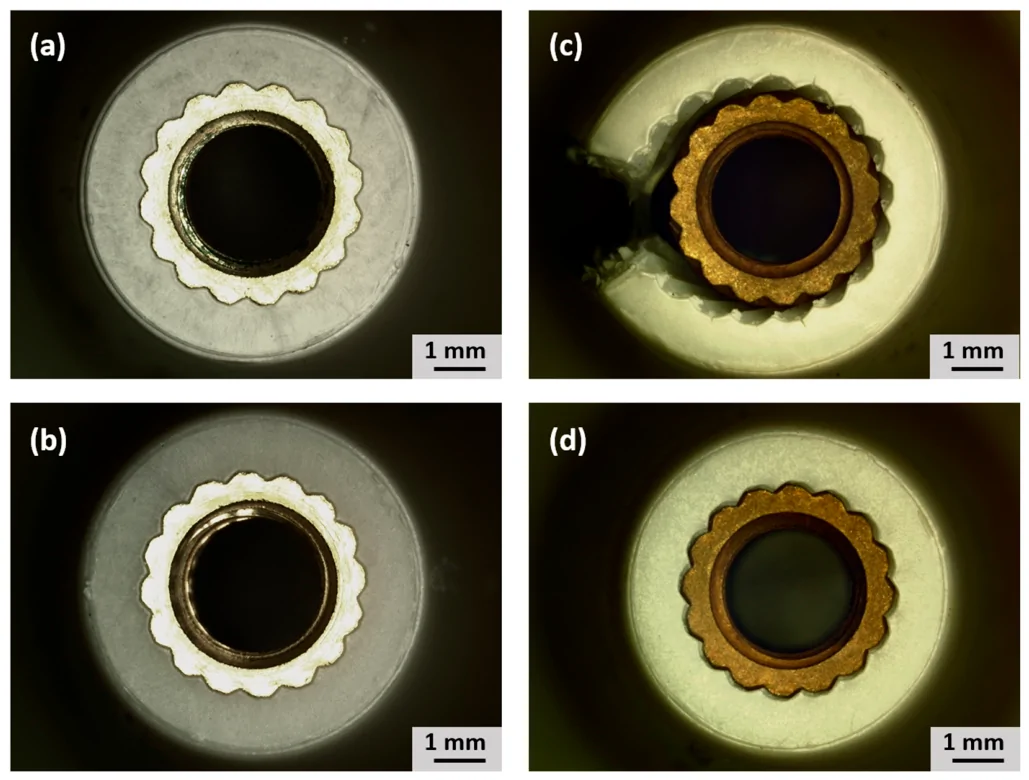
The surface morphologies of injection-molded screw boss specimens with embedded copper nuts, fabricated from conventional flame-retardant PC/ABS alloy with 7 wt.% MBS as toughener (**a**) and PC/ABS alloy with an optimized toughener system (**b**), and the surface morphologies of two samples after immersion in acetic acid for 12 h (**c**) and 168 h (**d**), respectively.

Inspired by the above findings, the strategy of constructing highly efficient crack-termination sites is extended to validate its universality in other modified PC systems. Conventional toughening agents used in modified PC materials like MBS (methacrylate-butadiene-styrene) with core–shell architectures (analogous to the coiled conformation in α-crystalline phase of PBT) exhibit limited crack-termination efficiency [35]. To achieve higher crack-termination efficiency, copolymers containing ethylene and acrylate segments were used to substitute MBS as the impact modifier (specific material compositions are shown in Table S1). These novel tougheners potentially form microcrystalline domains through ethylene chain alignment, mimicking the crack-termination mechanism of β-crystalline structures. As demonstrated in Figure 5a, even in BDP-flame-retarded PC/ABS alloys, which are notorious for their poor ESC resistance, replacing 7 wt.% MBS with ethylene-containing tougheners (including 5 wt.% PTW and 2 wt.% EMA) significantly enhances η from 0.23% to 1.17%. Furthermore, the severity of cracking was also significantly alleviated, as shown in Figure 5b–e; the PC/ABS sample changed from complete fracture to exhibiting only slight surface cracks after the ESC test. The microcrystalline regions within ethylene-containing tougheners create directional energy dissipation pathways, effectively disrupting crack propagation, leading to the significantly alleviated cracking of the sample with optimized tougheners. To validate practical applicability of this ESC optimizing strategy, an accelerated stress-releasing test was designed using injection-molded pillars with embedded copper nuts. This configuration amplifies stress concentration because of the difference in cooling shrinkage rate between copper nuts and polymer materials; even though there is currently no suitable characterization method to quantify the residual stress of non-transparent PC alloy in this structure, the ESC behavior of PC samples can still be compared under identical conditions [36,37,38]. These two samples made by BDP-flame-retarded PC/ABS alloys with 7 wt.% MBS and 7 wt.% ethylene-containing tougheners were immersed in acetic acid (99.9% purity), the conventional MBS-toughened flame-retarded PC/ABS cracked catastrophically within 12 h, while the optimized sample maintained structural integrity for 168 h, as shown in Figure 6.

The significant lifespan extension confirms that introducing highly efficient crack-termination sites can effectively improve the ESC resistance of PC-based materials. Furthermore, the strategy adopted in this work to improve the ESC resistance of the flame-retardant PC/ABS alloy by optimizing the toughener system will not significantly compromise the mechanical properties; as shown in Table 1, the reductions in tensile strength, flexural strength, flexural modulus, and notched impact strength are all within 5%. Moreover, even with these slight decreases, the mechanical properties of the optimized flame-retardant PC/ABS in this work remain superior to those of most PC/ABS alloys in the reported studies [39,40]. Furthermore, at the same flame-retardant (BDP) content of 12 wt.%, the flame retardancy of the material remained stable, maintaining a V-0 rating at a thickness of 2.0 mm. Furthermore, the ESC optimization strategy proposed in this work results in only a 5–10% increase in material cost, which, combined with the excellent retention of mechanical properties, indicates that this approach has good practical applicability.

## 4. Conclusions

In this work, we propose a universal strategy for enhancing the ESC resistance of PC and its alloys by introducing crack-termination sites. The crack-termination efficiencies of physical chain entanglements, rubber phases, inorganic fillers, and crystalline domains in PC samples were systematically evaluated. High-viscosity PC with high entanglement density, crystalline region of PBT and rubber phase of ABS and MBS can act as effective crack-termination sites. In contrast, inorganic fillers impair ESC resistance due to interfacial incompatibility and stress concentration. The β-crystalline phase in PC/PBT shows high crack-termination efficiency. Building on this insight, we extend the strategy to introduce ethylene-contained copolymers as tougheners of PC-based materials; long methylene segments in these tougheners induce microcrystalline regions during processing, which can effectively increase the critical strain rate (η) of the materials. This study provides a universal solution for improving the ESC resistance of PC and offers new insights for the development of high-performance modified PC materials.

## Figures and Tables

**Table 1:** Comparison of the mechanical performance and flame retardancy of the PC/ABS samples with conventional MBS toughener and the optimized toughener system in this work.

Properties	Test Standard	Condition	Sample	
			PC/ABS with MBS	PC/ABS with Optimized Toughener System
Tensile strength	ASTM D638	50 mm/min	60 Mpa	57 Mpa
Elongation at break	ASTM D638	50 mm/min	45%	51%
Flexural strength	ASTM D790	2 mm/min	89 Mpa	86 Mpa
Flexural modulus	ASTM D790	2 mm/min	2409 Mpa	2337 Mpa
IZOD impact strength, notched	ASTM D256	3.2 mm	760 J/m	730 J/m
Melt flow rate	ASTM D1238	260 °C/2.16 kg	14.1	12.3
Density	ASTM D792	23 °C	1.16	1.16
Flammability	UL 94	2.0 mm	V-0	V-0

## Data Availability

The data presented in this study are available from the corresponding author upon reasonable request.

## Supplementary Materials

The following supporting information can be downloaded at https://www.mdpi.com/article/10.3390/polym18172042/s1, Figure S1: DMA curves of PC samples with different viscosity; Figure S2: The morphological evolution of a medium-viscosity PC (PC 2100) specimen after sunscreen application at a strain of 1.5%: (a) 0 min; (b) 20 min; (c) 40 min; (d) 60 min; (e) 80 min; (f) 100 min. Under the action of environmental stress, the surface cracks continuously propagated and extended, eventually developing into through-thickness cracks that penetrated the entire specimen; Figure S3: SEM images of flame-retardant PC/ABS alloy with (a) 7 wt.% MBS as toughener and (b) an optimized toughener system (including 5 wt.% PTW and 2 wt.% EMA); Figure S4: The notched impact strength and critical strain rate of BDP-flame-retarded PC/ABS alloys incorporating different tougheners. The EMA toughener contains a higher proportion of polyethylene segments, so its compatibility with the PC/ABS matrix is inferior to that of the MBS toughener; as a result, the toughness of the flame-retardant PC/ABS decreased with increasing EMA content. Therefore, in this work, the PTW toughener (ethylene-butyl acrylate-glycidyl methacrylate terpolymer) containing reactive functional groups was adopted to enhance the compatibility between PC and EMA, thereby achieving higher toughening efficiency; Figure S5: The water absorption process of PC with different viscosities at room temperature; the viscosity of the PC resin had no significant effect on the final water uptake of the specimens; Figure S6: (a) Twin-screw extrusion process of the material; (b) the photo of material morphology after pelletizing; Figure S7: Images of the testing process: (a) DSC testing of different materials; (b) observation of crack morphology of the sample using a Leica optical microscope; (c) ESC testing of the sample based on the constant strain method used in this work; Figure S8. DSC curves of PC/PBT alloys with different content of PBT; According to the melting enthalpy, the crystallinity of the PC/40 wt.% PBT alloy is 15.4%, and that of the PC/30 wt.% PBT alloy is 10.9%. Figure S9: SEM image of the cross-section of PC/10 wt.% talc sample; Figure S10: FTIR spectra of PC/PBT alloys with and without transesterification inhibitor; Table S1: The specific material compositions of conventional flame-retardant PC/ABS alloy and the PC/ABS alloy with an optimized toughener system; Table S2: The critical strain rate (η) of different samples upon exposure to different solvents for 168 h.
