# Supplementary material for: Introducing Crack–Termination Sites to Improve the Resistance of Polycarbonate on Environmental Stress Cracking
Source: Polymers (Basel). 2026 Aug 23;18(17):2042. doi: 10.3390/polym18172042 (PMC13567991; doi:10.3390/polym18172042)
Supplement: Supplementary file 1 [file polymers-18-02042-s001.zip › polymers-4490145-supplementary.pdf]

Supporting Information

Introducing crack termination sites to improve the resistance of polycarbonate on environmental stress cracking

*Minjian Ma[1,2], Qian Huang[1], Peitao Wang[2], Junwei Ai[2], Huiqiang Liang[2], Liang Yu[2], Minle Peng[2], Yin Cen[2,3]**

[1]Key Laboratory of Polymer Materials Engineering, Polymer Research Institute, Sichuan University, 610065 Chengdu, China

[2]Kingfa Science and Technology, Co., Ltd., Guangzhou 510663, China

[3]Huazhong University of Science and Technology, Wuhan 430074, China

*Correspondence: ceny@kingfa.com.cn;

Keywords: Polycarbonate, environmental stress cracking, crack termination sites, critical strain rate

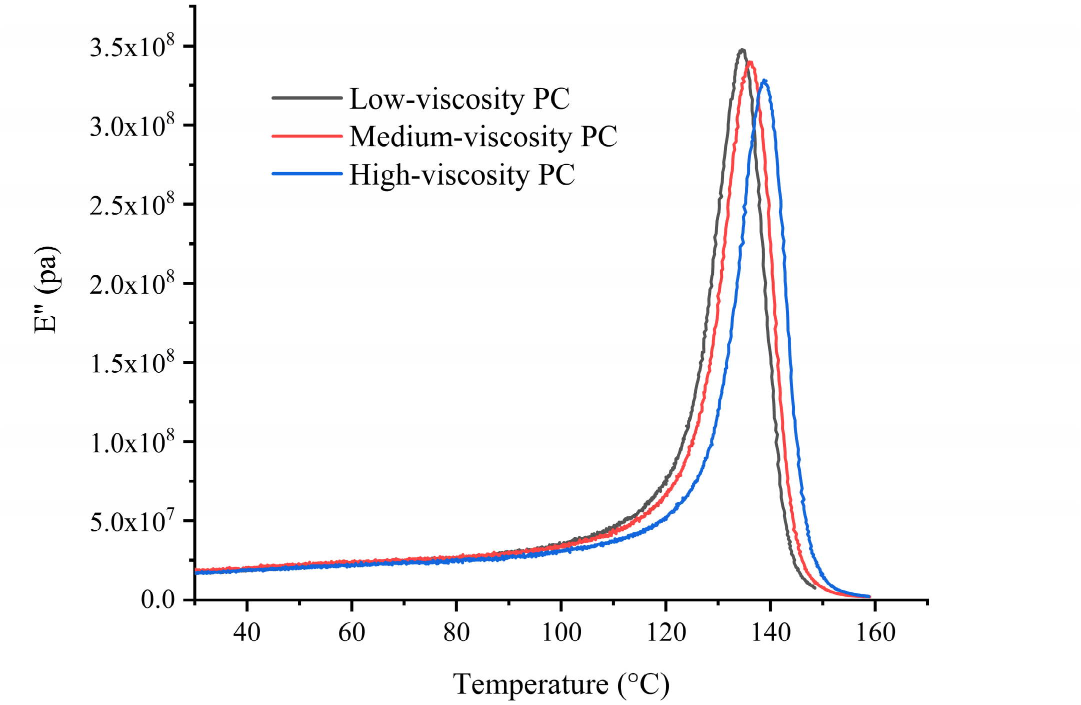

Figure S1. DMA curves of PC samples with different viscosity.

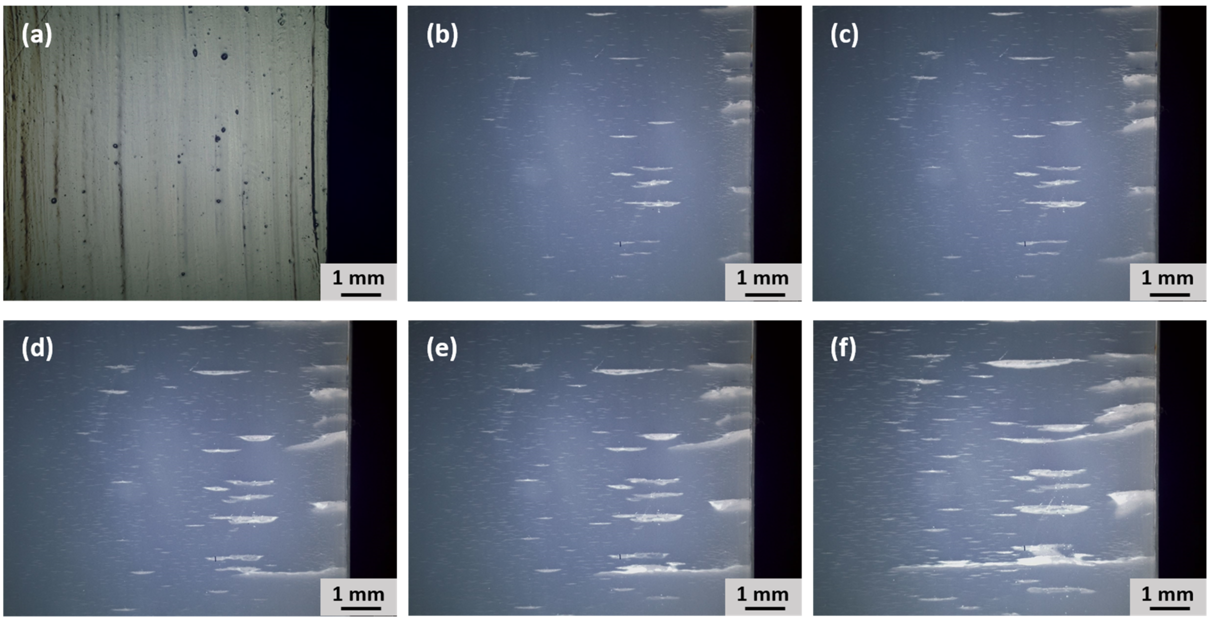

Figure S2. The morphological evolution of a medium-viscosity PC (PC 2100) specimen after sunscreen application at a strain of 1.5%: (a) 0 min; (b) 20min; (c) 40min; (d) 60min; (e) 80min; (f) 100min. Under the action of environmental stress, the surface cracks continuously propagated and extended, eventually developing into through-thickness cracks that penetrated the entire specimen.

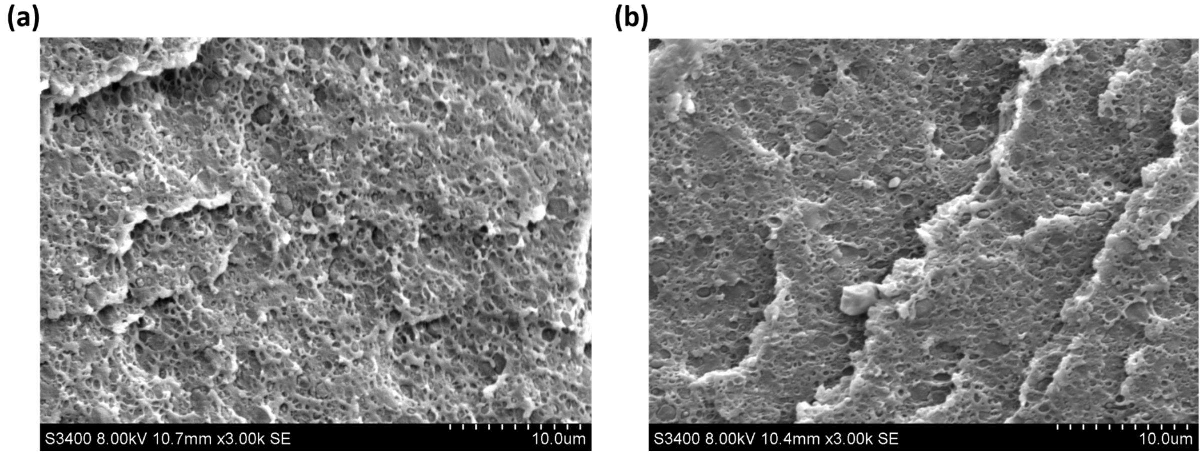

Figure S3. SEM images of flame-retardant PC/ABS alloy with (a) 7wt.% MBS as toughener and (b) the an optimized toughener system (include 5 wt.% PTW and 2wt.% EMA)

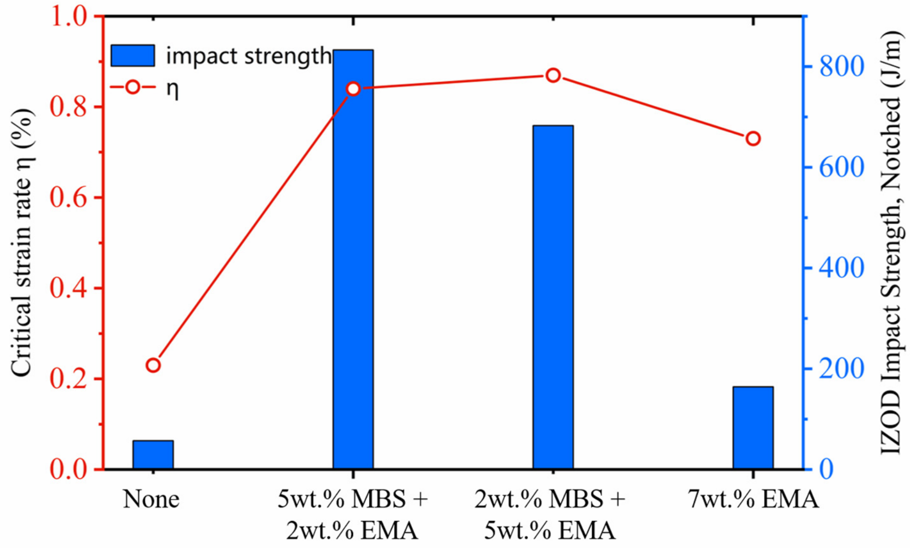

Figure S4. The notched impact strength and critical strain rate of BDP-flame-retarded PC/ABS alloys incorporating different tougheners. The EMA toughener contains a higher proportion of polyethylene segments, so its compatibility with the PC/ABS matrix is inferior to that of the MBS toughener; as a result, the toughness of the flame-retardant PC/ABS decreased with increasing EMA content. Therefore, in this work, the PTW toughener (ethylene-butyl acrylate-glycidyl methacrylate terpolymer) containing reactive functional groups was adopted to enhance the compatibility between PC and EMA, thereby achieving a higher toughening efficiency.

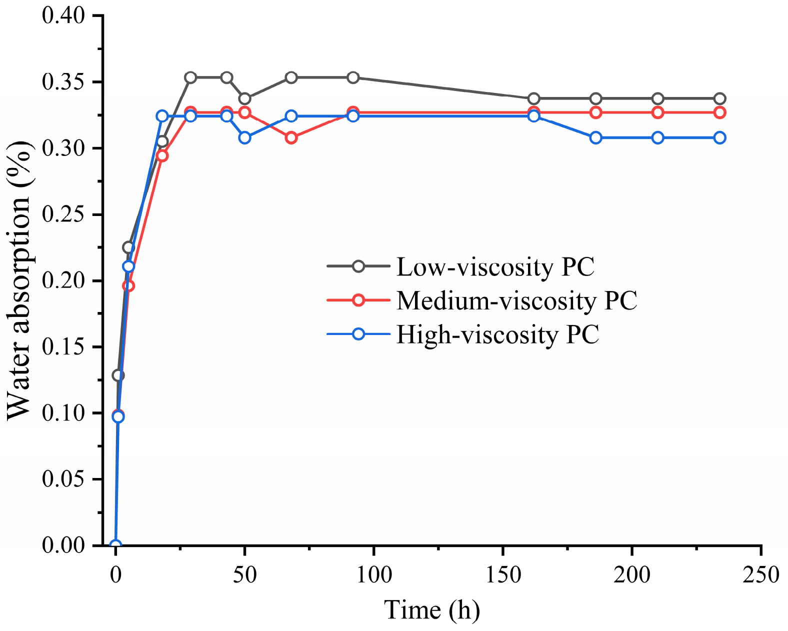

Figure S5. The water absorption process of PC with different viscosities at room temperature, the viscosity of the PC resin had no significant effect on the final water uptake of the specimens.

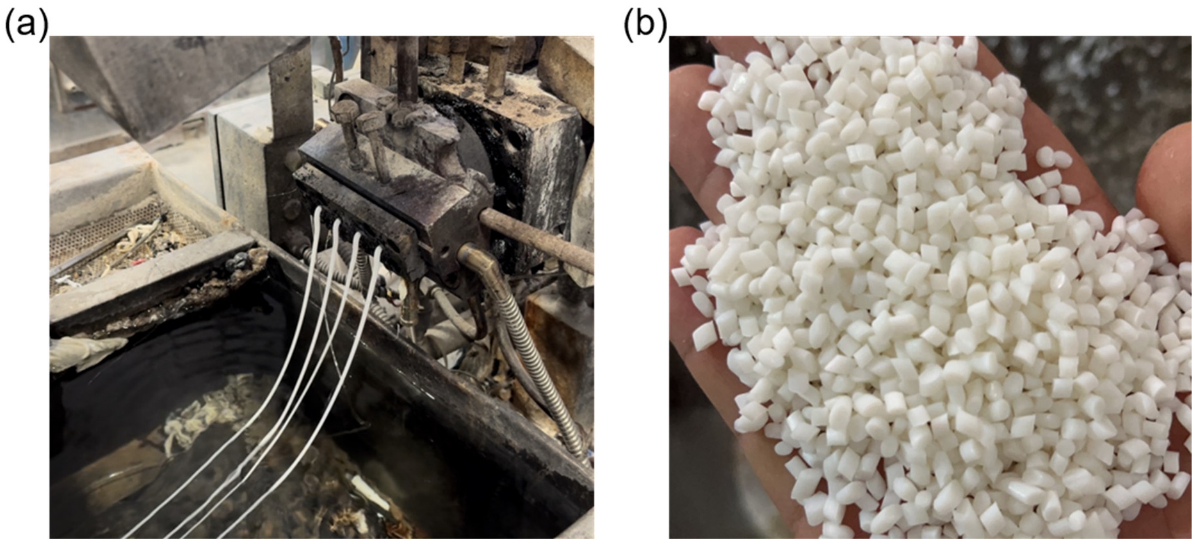

Figure S6. (a) Twin-screw extrusion process of the material; (b) The photo of material morphology after pelletizing.

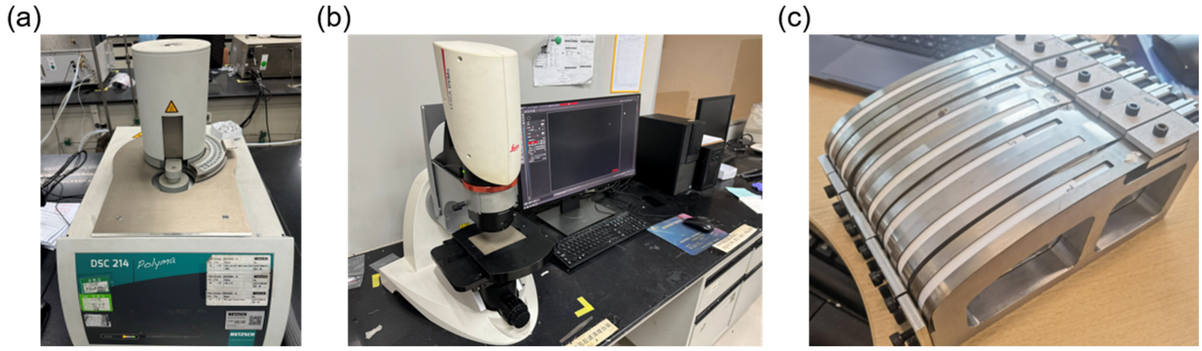

Figure S7. Images of the testing process: (a) DSC testing of different materials; (b) Observation of crack morphology of the sample using a Leica optical microscope; (c) ESC testing of the sample based on the constant strain method used in this work.

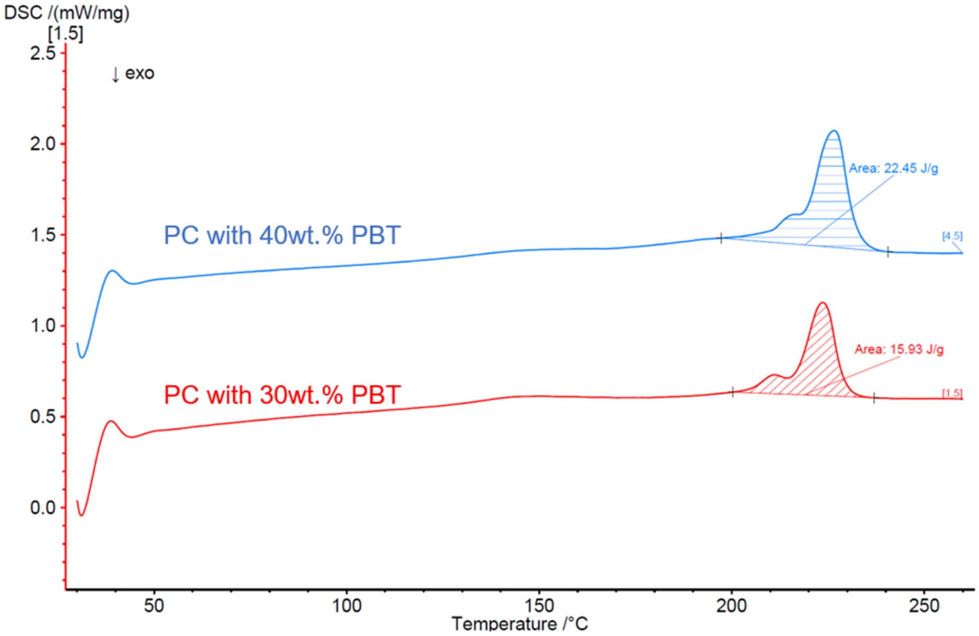

Figure S8. DSC curves of PC/PBT alloys with different content of PBT; According to the melting enthalpy, the crystallinity of the PC/40 wt.% PBT alloy is 15.4%, and that of the PC/30 wt.% PBT alloy is 10.9%.

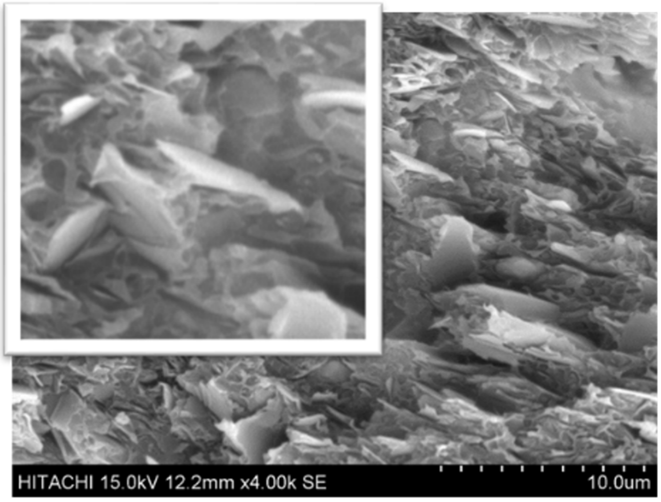

Figure S9. SEM image of the cross-section of PC/10wt.% talc sample.

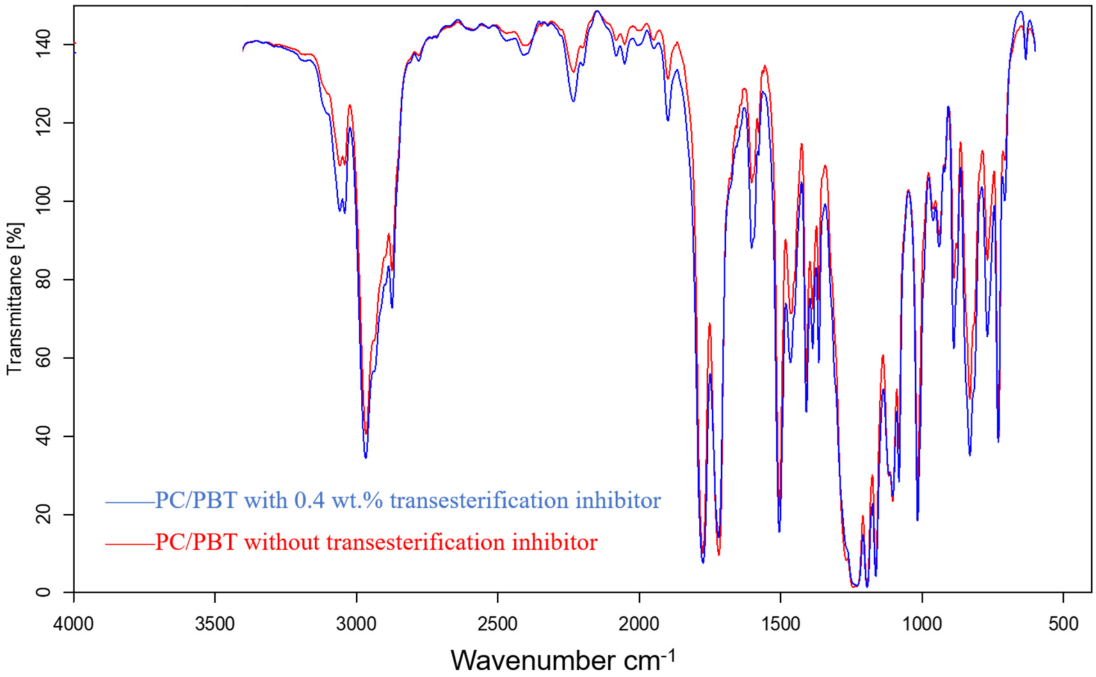

Figure S10. FTIR spectra of PC/PBT alloys with and without the transesterification inhibitor.

Table S1 The specific material compositions of conventional flame-retardant PC/ABS alloy and the PC/ABS alloy with an optimized toughener system.

Composition	Conventional flame-retardant PC/ABS alloy	Flame-retardant PC/ABS alloy with an optimized toughener system
PC 2100	68.5 wt.%	68.5 wt.%
ABS 8434	12 wt.%	12 wt.%
BDP (FP-600)	12 wt.%	12 wt.%
MBS (EM500)	7 wt.%	
EMA (Elvaloy AC resin 1125)		2 wt.%
PTW		5 wt.%
Processing additives	0.5 wt.%	0.5 wt.%

Table S2 The critical strain rate (η) of different samples upon exposure to different solvents for 168h;

Solvent	Conventional flame-retardant PC/ABS alloy	Flame-retardant PC/ABS alloy with an optimized toughener system
Ethanol	$\eta = 1.43\%$	No cracking
sodium hypochlorite	$\eta = 0.61\%$	$\eta = 1.20\%$
Peanut oil	$\eta = 0.89\%$	$\eta = 1.41\%$
